# Serum C-reactive protein concentration in early abdominal and pulmonary sepsis

**DOI:** 10.1186/cc12669

**Published:** 2013-06-19

**Authors:** JA Orati, SM Lobo

**Affiliations:** 1 University of Ribeirão Preto, SP, Brazil; 2 Hospital de Base de São José do Rio Preto, SP, Brazil

## Introduction

The objective of this study was to evaluate the C-reactive protein serum levels in patients with pulmonary and abdominal sepsis during the first 5 days of sepsis progression.

## Methods

The present investigation was a retrospective cohort study conducted at the university hospital with 345 patients who were admitted to the ICU and diagnosed with sepsis of pulmonary or abdominal origin. Serum C-reactive protein concentrations were measured by the turbidimetric immunoassay. For analysis of C-reactive protein, day 1 was defined as the day on which the patient was clinically diagnosed with sepsis.

## Results

Thirty-four patients with sepsis (9.8%), 114 patients with severe sepsis (33.0%), and 197 patients with septic shock (57.2%) were evaluated. The age of the patients was 56.4 ± 19.8 years. The serum C-reactive protein concentrations were higher on the day of sepsis diagnosis in the group with abdominal infection compared with the group with pulmonary sepsis (17.8 ± 10.1 mg/dl vs. 14.9 ± 11.1 mg/ dl, *P *= 0.025) and remained significantly higher during the first 5 days of sepsis progression. The values of the area under the ROC curve, sensitivity, specificity, and best cutoff points are listed in Table 1.

**Table 1:** Values of the area under the ROC curve, sensitivity, specificity, and best cutoff points

ROC curve	AUC (95% CI)	Sensitivity	Specificity	Cutoff (mg/dl)
CRP day 1	0.53 (0.43 to 0.62)	0.26	0.88	6.12
CRP day 2	0.51 (0.41 to 0.60)	0.67	0.42	18.6
CRP day 3	0.52 (0.43 to 0.61)	0.57	0.56	13.6
CRP day 4	0.51 (0.41 to 0.60)	0.42	0.65	14.2
CRP day 5	0.59 (0.48 to 0.68)	0.58	0.62	9.10

AUC, area under the ROC curve; CI, confidence interval; CRP, C-reactive protein; ROC, receiver operator characteristic.

## Conclusion

The accuracy of C-reactive protein for the differential diagnosis of pulmonary and abdominal sepsis is limited, although significantly higher serum levels were observed in patients with abdominal sepsis.

